# Academic Burnout and Problematic Smartphone Use During the COVID-19 Pandemic: The Effects of Anxiety and Resilience

**DOI:** 10.3389/fpsyt.2021.725740

**Published:** 2021-10-20

**Authors:** Zejun Hao, Liangyi Jin, Jinzi Huang, Ruibo Lyu, Qian Cui

**Affiliations:** ^1^Institute of Foreign Languages, China Medical University, Shenyang, China; ^2^Department of Obstetrics, Shenyang Women's and Children's Hospital, Shenyang, China; ^3^Department of Preschool Education, Liaoning National Normal College, Shenyang, China; ^4^Faculty of Psychology, Beijing Normal University, Beijing, China; ^5^Department of Rehabilitation, Shengjing Hospital of China Medical University, Shenyang, China

**Keywords:** anxiety, resilience, problematic smartphone use, academic burnout, COVID-19

## Abstract

**Background:** Academic burnout has been associated with problematic smartphone use. However, the mechanism underlying this relation has been inadequately explored during the COVID-19 pandemic.

**Materials and Methods:** A total of 748 Chinese undergraduate students were recruited in the study who were measured with their levels of academic burnout, anxiety, resilience, and problematic smartphone use.

**Results:** Our study showed that academic burnout significantly predicted problematic smartphone use both directly and indirectly via anxiety. By constructing a moderated mediation model, our study found that resilience moderated the direct impact and the second half of the indirect path (between anxiety and problematic smartphone use); however, with the moderation effects of resilience, both the indirect impact of academic burnout on problematic smartphone use *via* anxiety became insignificant.

**Conclusions:** Our findings brought additional evidence on the association between academic burnout and problematic smartphone use and significantly suggested the potential solution to alleviate the influences.

## Introduction

The advancements in communication technology have greatly extended the scenarios where the smartphone has become an integral part of daily routine. As of December 2020, the number of smartphone internet users has reached 989 million in China (1). Besides the benefits of instant communication and enhanced productivity, researchers have reported adverse outcomes due to excessive smartphone use and have suggested the predicting effect of psychopathology. During the COVID-19 pandemic, home quarantine and altered teaching style have increased the distress level of college students (2, 3), which could potentially disrupt their academic work. The current study focused on the association between academic burnout and problematic smartphone use; in addition, we examined the mediation effect of anxiety and the moderation effects of resilience in this relation.

### Problematic Smartphone Use

Problematic smartphone use describes the maladaptive use of smartphone with functional impairments on the users (4, 5). Although uncontrollable smartphone use has displayed some similar symptoms with those of addictive behaviors (6), considering it has not been officially classified in either DSM-5 or ICD-11, researchers keep cautious about using the term “addiction.” Accordingly, we used problematic smartphone use in our study. Problematic smartphone use is linked with negative psychological outcomes of loneliness (7), interpersonal problems (8), and lower level of altruism (9). In addition, individuals with problematic smartphone use are more likely to have sleep disorder (10), hand dysfunction (11), and shoulder pain (12). For college students, problematic smartphone use is associated with declined academic performance (13). Some studies have proposed that the major motivation for conducting smartphone overuse is to release the negative emotions (14) and have come up with the predictors of depression and anxiety during the COVID-19 pandemic (15, 16). However, the impact of academic burnout on problematic smartphone use under the status quo has been insufficiently explored.

### Academic Burnout and Problematic Smartphone Use

Academic burnout, which stems from job burnout, is defined as a trifactorial construct characterized by symptoms of emotional exhaustion (feeling exhausted due to study work and demand), cynicism (indifferent to college degree and peers), and academic inefficacy (low personal academic achievement) (17, 18). Academic burnout is driven by the constant exposure to internal and external stressors (19, 20) and adversely influences the well-being. Students with academic burnout are subject to decline in academic performances (21) and are more likely to have sleep disorders (22), fatigue (23), depressive symptoms (24), and low self-esteem (25). In addition, academic burnout is associated with addictive behaviors such as substance abuse (26) and eating disorder (27). Given these, individuals as such are more likely to overuse their smartphones. To relieve emotional exhaustion and frustration due to academic inefficacy, a student with academic burnout would turn to smartphone use for relaxation; however, when this intention becomes out of control, problematic smartphone use would be intrigued (28).

### The Potential Mediation Effect of Anxiety

Another psychological factor related to academic burnout is anxiety. Anxiety is the psychological response of an individual to the challenging situation (29). According to the previous studies, an individual with more burnout symptoms is prone to a higher anxiety level. This could be explained by the emotional exhaustion and cynical components of burnout that would intrigue the protective mechanism within the body and increase the level of anxiety of an individual (30, 31). In addition, an individual with anxiety would more likely use addictive behaviors to get rid of the negative emotions. This is supported by the findings that anxiety is a significant predictor of problematic smartphone use (15, 32). Based on these, we hypothesized that anxiety would be a mediator between academic burnout and problematic smartphone use.

### The Potential Moderation Effects of Resilience

Resilience describes the capability of an individual to bounce back in face of failures and tough situations (33). Resilience, as a positive factor, is linked with better psychological outcomes. An individual with a higher level of resilience would be more capable of dealing with the influences brought by the negative emotions (34). In addition, resilience, as an effective coping strategy, could give an individual some thoughtful insights on the status quo and enables one to get through in good time. These would help an individual to keep off the possibilities of conducting addictive behaviors (35).

### The Theory

A comprehensive theory for explaining the progression of problematic smartphone use is the Interaction of Person–Affect–Cognitive–Execution model (I-PACE) (36, 37). The I-PACE model has proposed the predisposing factors of problematic smartphone use such as personal traits, specific needs, psychopathology, etc. This model has also demonstrated the underlying mechanism that the predisposing factors would first intrigue the affective and cognitive responses within an individual, and then all these determinants combined determine the problematic smartphone use. In addition, the I-PACE model has suggested the moderation effects of certain coping strategies in the progression of problematic smartphone use. In the current study, we conceptualized the academic burnout as the psychopathological factor and anxiety as the affective response, which mediated between academic burnout and problematic smartphone use; in addition, resilience was hypothesized as a cope strategy to moderate the progression of problematic smartphone use.

### The Current Study

**Figure 1:**
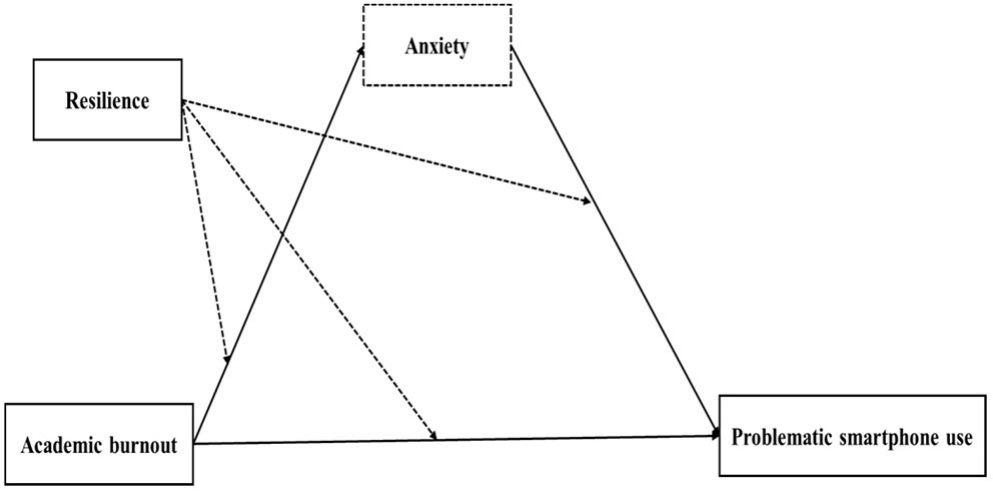
The hypothesized model. The dotted boxes contained the investigated mediator and the dotted lines indicated the investigated moderation effects.

The current study aimed to explore the mediation effect of anxiety and the moderation effects of resilience in the association between academic burnout and problematic smartphone use. Our hypotheses were as follows: (1) Academic burnout positively predicted problematic smartphone use. (2) Anxiety mediated this association. (3) Resilience moderated both the direct and the indirect impacts of academic burnout on problematic smartphone use. The hypothesized model is depicted in Figure 1.

## Methods

### Participants and Procedure

The participants were recruited from two universities in Northeast China by the method of stratified random cluster sampling. To guarantee the diversity of participants, the undergraduate students participating in this study majored in various disciplines including Linguistics, Education of Art, Music, Engineering, etc. All the participants were registered Chinese undergraduate students who had the full capability to read and understand the survey questions and were competent to complete the survey independently. The online survey took place in May 2020. Due to the COVID-19 pandemic, all the students were subject to home quarantine and joined in online classrooms. Digital questionnaires were saved and forwarded to the participants at the end of the online classes. The survey took 20 min. The participants clicked the submit button, and answers were collected automatically. Of the 822 individuals enrolling in the study, 74 participants were excluded because their responses contained unanswered questions (resulting effective *N* = 748). The average age of the participants was 20.12 years (SD = 1.14). More female students (*n* = 570, 76.2%) joined in the survey than male students (*n* = 178, 23.8%). The survey was approved by the Ethics Committee of Liaoning National Normal College, and a written consent was obtained from each participant. Small gifts of key chains were given to participants for joining this survey.

### Measures

#### Smartphone Addiction Scale—Short Version

We used the smartphone addiction scale—short version (SAS-SV) (38) to measure the levels of problematic smartphone use among the participants. It consists of 10 items and runs on a six-point Likert system ranging from 1 = strongly disagree to 6 = strongly agree. Samples items include, e.g., “Missing planned work due to smartphone use” and “Having a hard time concentrating in class, while doing assignments, or while working due to smartphone use.” The higher scores obtained indicate the higher levels of problematic smartphone use. SAS-SV has been widely used and proved with great reliability among Chinese participants (16, 32). Cronbach's alpha in the current sample was 0.814.

#### Learning Burnout Scale

The levels of academic burnout of the participants were measured by the learning burnout scale (LBS) (39), which was revised on the basis of the Maslach Burnout Inventory (40). The scale, which was specifically designed for Chinese students, consists of 20 items and runs on a five-point Likert system ranging from 1 = strongly disagree to 5 = strongly agree. Sample items include, e.g., “I seldom study after school” and “I feel exhausted when studying.” The higher scores obtained indicate the higher levels of academic burnout. It has been widely used and proved with good validity and reliability for Chinese students (39, 41). The Cronbach's alpha in the current sample was 0.862.

#### Depression, Anxiety, Stress Scale-21

The depression, anxiety, stress scale-21 (DASS-21) (42), which contains 21 items has been widely used to measure the levels of depression, anxiety, and stress symptoms. As suggested by previous studies (42, 43), we used the anxiety subscale of DASS-21 to measure the level of anxiety over the past week. This subscale has seven items and is based on a four-point Likert system ranging from 0 = did not apply to me to 3 = applied to me very much. Sample items include, e.g., “I was aware of dryness of my mouth” and “I experienced trembling (e.g., in the hands).” The higher scores obtained indicate the higher level of anxiety symptom. The Cronbach's alpha of anxiety subscale in the current sample was 0.842.

#### The Connor–Davidson Resilience Scale

The Connor–Davidson Resilience Scale (CD-RISC) (33) was used to measure the levels of resilience. It includes 25 items and runs on a five-point Likert system, ranging from 0 = not true at all to 4 = true nearly all the time. Sample items include, e.g., “I am able to adapt when changes occur” and “Tend to bounce back after illness or hardship.” The higher scores obtained indicate a higher level of resilience. The questionnaire has been widely used for measuring resilience among Chinese population with good validity (44, 45). The Cronbach's alpha in the current sample was 0.929.

### Statistical Analyses

SPSS 22.0 and SPSS macro PROCESS 3.1 (46) were used for statistical analyses in this study. The significant level was set at 0.05. First, we checked the distribution of the data and found that they were in a non-normal distribution. Spearman analysis was conducted to examine the correlations of all the investigated variables in this study. Second, as suggested by Preacher et al. and Hayes (47, 48), the analyses *via* the SPSS PROCESS macro had no requirement on the distribution of the data. Thus, model four of SPSS macro PROCESS 3.1 (46) was used to test the mediation effect of anxiety in the relationship between academic burnout and problematic mobile phone use by generating bias-corrected bootstrap confidence interval (using 5,000 bootstrapping samples). Third, a moderated mediation model was constructed with model 59 of SPSS macro PROCESS 3.1 (46) to test the mediation effect of anxiety and the moderation effects of resilience between academic burnout and problematic smartphone use by generating bias-corrected bootstrap confidence interval (using 5,000 bootstrapping samples). Given the differences of age and gender in problematic mobile phone use (6, 49), age and gender were treated as controlled variables in the present study.

## Results

### Correlations of the Investigated Variables

The results of the Spearman analysis showed that all the investigated variables were in significant association with each other. Academic burnout is positively associated with anxiety and problematic smartphone use while negatively correlated with resilience (see Table 1).

**Table 1:** Correlations of the investigated variables.

	**1**	**2**	**3**	**4**
1. Academic burnout				
2. Anxiety	0.367^***^			
3. Resilience	−0.336^***^	−0.2^***^		
4. PSU	0.348^***^	0.234^***^	−0.502^***^	
Medians	49	6	87	30
P_25_, P_75_	42, 58	2, 12	78, 97	25, 35

N = 748, ^***^p < 0.001. PSU, problematic smartphone phone use; P_25_, 25^th^ percentile; P_75_, 75^th^ percentile. The data were in non-normal distribution and spearman analysis was conducted accordingly.

### Testing for the Mediation Effect

The results of the mediation analyses showed that after controlling for age and gender, academic burnout positively predicted problematic mobile phone use both directly (β = 0.281, *p* < 0.001) and indirectly via anxiety (β = 0.028, 95% confidence interval 0.007–0.054) (see Tables 2, 3). Namely, anxiety partially mediated the impact of academic burnout on problematic mobile phone use.

**Table 2:** Mediation analysis.

**Outcome variables**	**Independent variables**	**β**	**SE**	***t***	***P*** **-value**
PSU	Constant	17.377^***^	5.337	3.256	=0.001
	Age	−0.189	0.245	−0.772	0.44
	Gender^a^	−0.301	0.658	−0.458	0.647
	Academic burnout	0.309^***^	0.029	10.836	<0.001
Anxiety	Constant	−2.754^***^	4.292	−0.642	0.521
	Age	0.117	0.197	0.593	0.554
	Gender^a^	−1.607^**^	0.529	−3.036	<0.01
	Academic burnout	0.233^***^	0.023	10.14	<0.001
PSU	Constant	17.712^***^	5.317	3.331	=0.001
	Age	−0.203	0.244	−0.833	0.405
	Gender^a^	−0.106^*^	0.66	−0.161	0.872
	Anxiety	0.121^**^	0.045	2.676	<0.01
	Academic burnout	0.281^***^	0.03	9.266	<0.001

*N = 748,*

* *p < 0.05,*

** *p < 0.01,*

*** *p < 0.001.*

*PSU, Problematic smartphone use;*

a *male = 1, female = 2*.

**Table 3:** Bootstrapping indirect effect and 95% confidence interval (CI) for the mediation test.

**Indirect path**	**Estimated effect**	**95% CI**	
		**BootLLCI**	**BootULCI**
Academic burnout → anxiety → PSU	^a^0.028	0.007	0.054

*N = 748; Bootstrap sample size, 5,000; PSU, Problematic smartphone use; LL, low limit; CI, confidence interval; UL, upper limit.*

a *Empirical 95% confidence interval does not overlap with zero*.

### Testing for the Moderated Mediation Model

**Figure 2:**
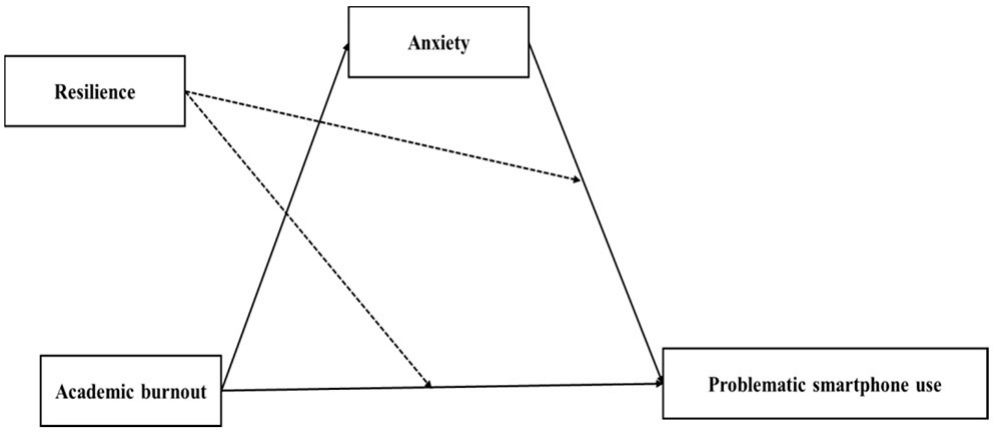
The verified model of between academic burnout and problematic smartphone use (*N* = 748). The study found that acadmic burnout positively predicted problematic smartphone use both directly and indirectly *via* anxiety. Resilience significantly moderated between academic burnout and problematic smartphone use and between anxiety and problematic smartphone use.

**Figure 3:**
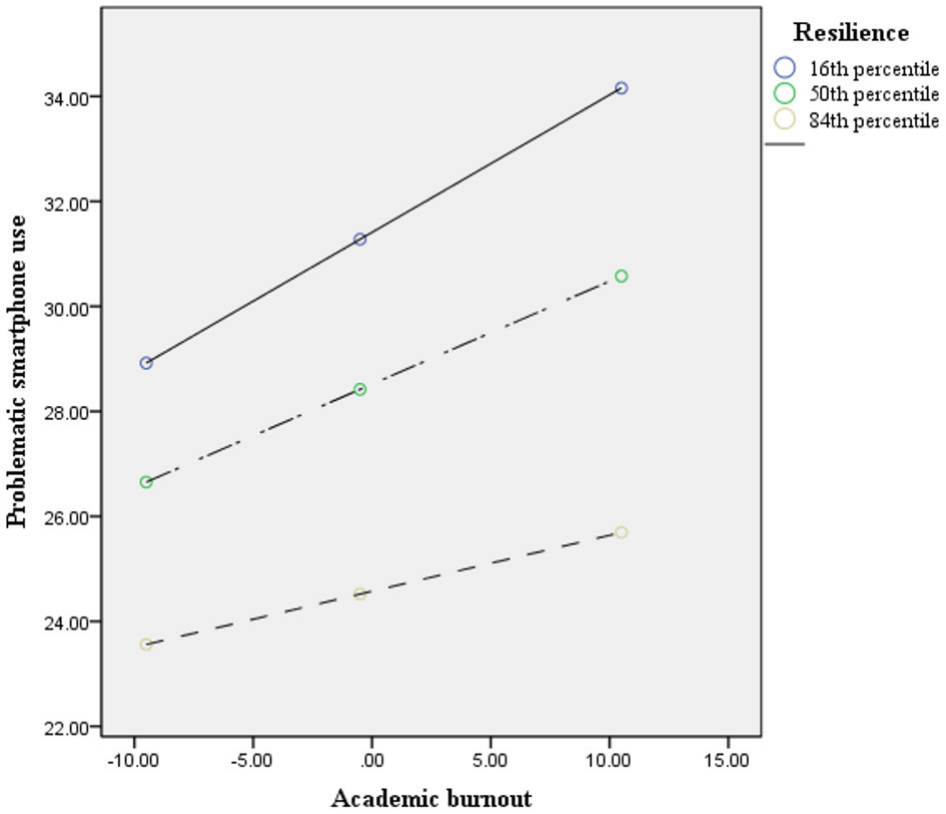
The conditional effect between academic burnout and problematic smartphone use (*N* = 748). To further understand the moderation effect of resilience on the path between academic burnout and problematic smartphone use, a conditional indirect effect analysis was conducted at 16th, 50^th^, and 84th percentiles resilience.

**Figure 4:**
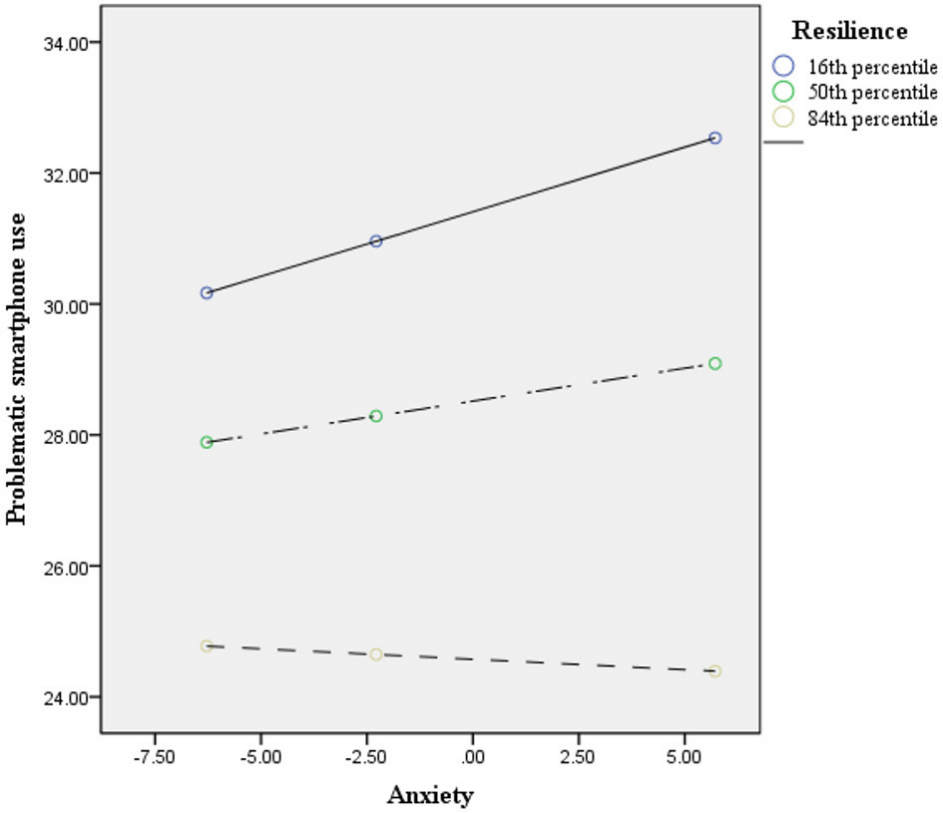
The conditional effect between anxiety and problematic smartphone use (*N* = 748). To further understand the moderation effect of resilience on the path between anxiety and problematic smartphone use, a conditional indirect effect analysis was conducted at 16th, 50^th^, and 84th percentiles resilience.

**Figure 5:**
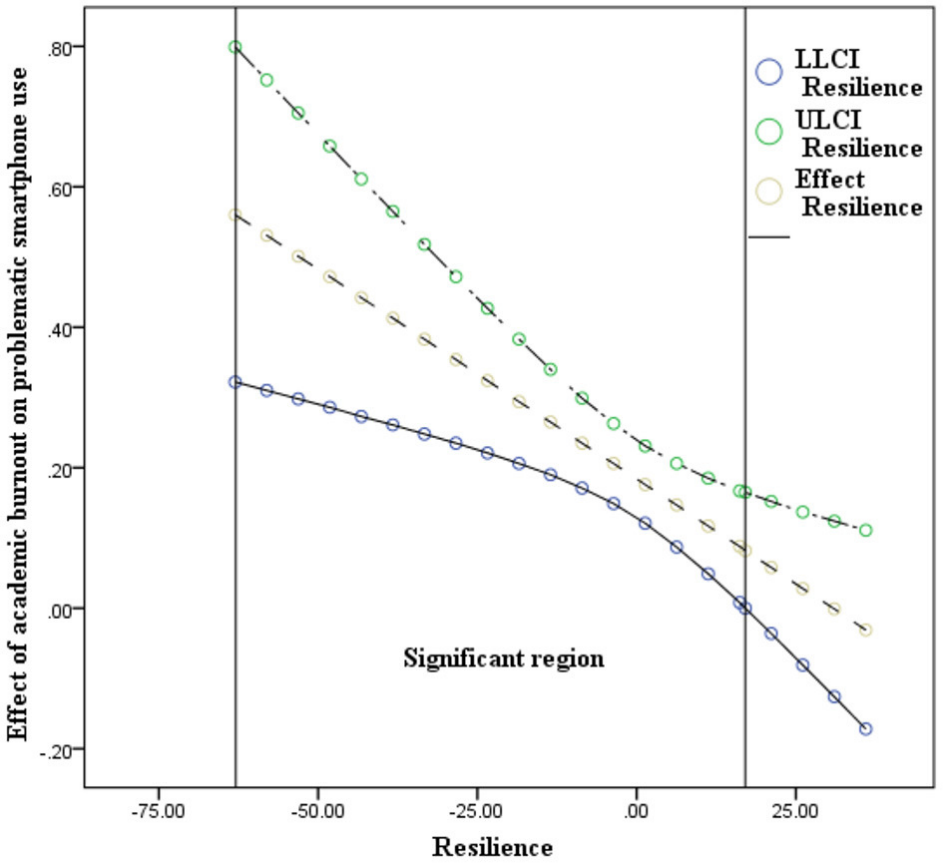
Effects of resilience with 95% confidence interval (*N* = 748). LL, low limit; CI, confidence interval; UL, upper limit. The Johnson-Neyman (J-N) method was applied to measure the significant region in which resilience significantly moderated the impact of academic burnout on problematic smartphone use. The upper and lower lines indicated the limits of confidence interval and the middle line indicated the effect of resilience. The scores of statistical significance ranged from −63.027 to 17.053.

**Figure 6:**
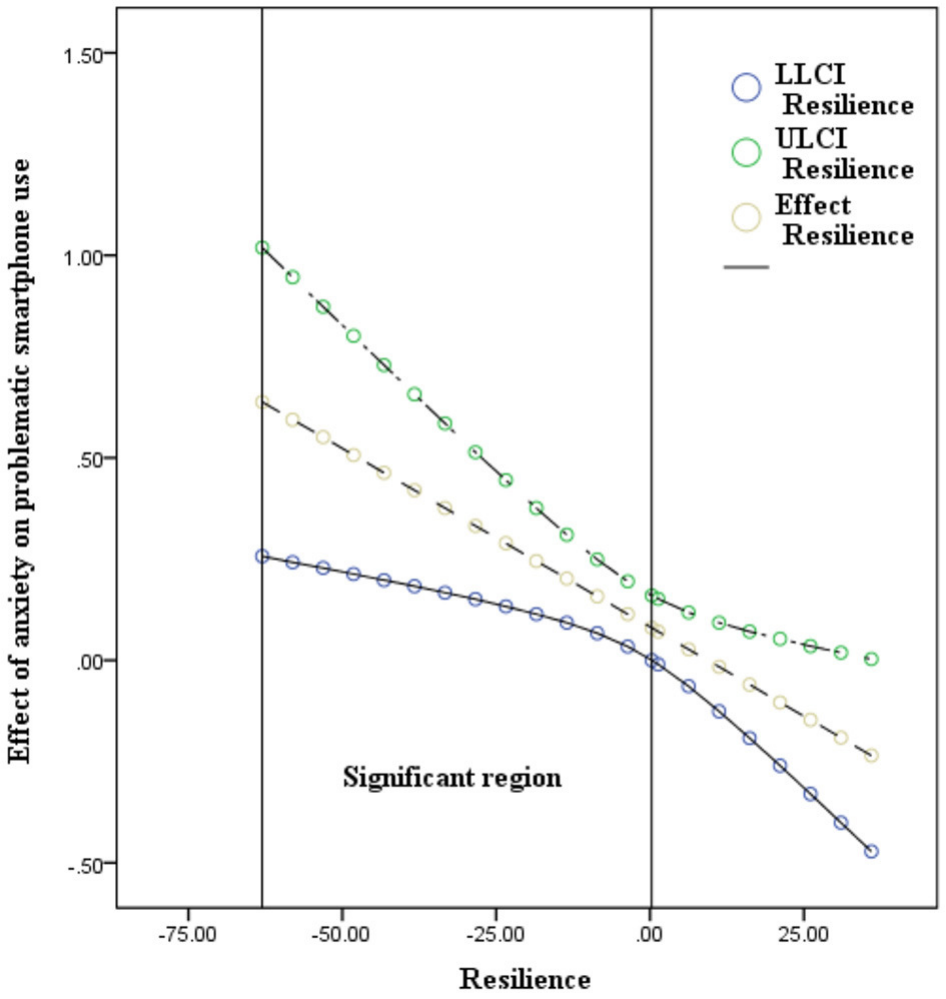
Effects of resilience with 95% confidence interval (*N* = 748). LL, low limit, CI, confidence interval, UL, upper limit. The Johnson-Neyman (J-N) method was applied to measure the significant region in which resilience significantly moderated the impact of anxiety on problematic smartphone use. The upper and lower lines indicated the limits of confidence interval and the middle line indicated the effect of resilience. The scores of statistical significance ranged from −63.027 to 0.287.

The results of model 59 of SPSS macro PROCESS 3.1 (46) are shown in Tables 4, 5. After controlling for age and gender, the interaction of academic burnout and resilience had a significant effect on problematic smartphone use: with a higher level of resilience, the positive association between academic burnout and problematic smartphone use became less positive (academic burnout × resilience, β = −0.006, *p* = 0.001, 95% confidence interval −0.01 to −0.002); the interaction of anxiety and resilience had a significant effect on problematic smartphone use: with a higher level of resilience, the positive association between anxiety and problematic smartphone use became less positive (anxiety × resilience, β = −0.009, *p* < 0.01, 95% confidence interval −0.015 to −0.003); however, the interaction of academic burnout and resilience had no significant effect on anxiety: (academic burnout × resilience, β = 0.001, *p* = 0.434, 95% confidence interval −0.002–0.004). Namely, resilience significantly moderated between academic burnout and problematic smartphone use, and between anxiety and problematic smartphone use. However, the moderated mediation model was not supported. With the moderation effects of resilience, academic burnout only directly predicted problematic smartphone use (16th percentile of resilience = −13.027, β = 0.262, 95% confidence interval 0.188–0.336; 50th percentile of resilience = −2.027, β = 0.196, 95% confidence interval 0.14–0.252; 84th percentile of resilience = 12.973, β = 0.107, 95% confidence interval 0.035–0.179). There was no mediation effect of anxiety between academic burnout and problematic smartphone use with the moderation effects of resilience (16th percentile of resilience = −13.027, β = 0.04, 95% confidence interval 0.012–0.069; 50th percentile of resilience = −2.027, β = 0.022, 95% confidence interval 0.002–0.042; 84th percentile of resilience = 12.973, β = 0.008, 95% confidence interval −0.035–0.016) (see Figure 2). To better understand each individual moderation effect of resilience, the analyses were conducted at the 16th, 50^th^, and 84th percentiles, and the results are depicted in Figures 3, 4. For a better display of the statistically significant region or the range of scores in which resilience moderated, the Johnson–Neyman (J-N) method was applied (see Figures 5, 6). The analyses showed that the resilience scores of statistical significances ranged from −63.027–17.053 between academic burnout and problematic smartphone use and from –63.027–0.287 between anxiety and problematic smartphone use.

**Table 4:** Moderated mediation analysis by model 59.

**Outcome variables**	**Independent variables**	**β**	**SE**	**t**	***P*** **-value**
Anxiety	Constant	0.276	4.104	0.067	0.946
	Age	0.14	0.198	0.707	0.48
	Gender^a^	−1.721^***^	0.532	−3.234	=0.001
	Academic burnout	0.219^***^	0.024	9.046	<0.001
	Resilience	−0.029	0.018	−1.674	0.095
	Academic burnout x resilience	0.001	0.002	0.784	0.434
PSU	Constant	33.03^***^	4.521	7.306	<0.001
	Age	−0.175	0.218	−0.802	0.423
	Gender^a^	−0.869	0.591	−1.471	0.142
	Academic burnout	0.184^***^	0.028	6.551	<0.001
	Anxiety	0.082^*^	0.041	2.03	<0.05
	Resilience	−0.263^***^	0.19	−13.49	<0.001
	Academic burnout x resilience	−0.006^***^	0.002	−3.201	=0.001
	Anxiety x resilience	−0.009^**^	0.003	−2.871	<0.01

*N = 748,*

* *p < 0.05,*

** *p < 0.01,*

*** *p < 0.001. Bootstrap sample size, 5,000; PSU, Problematic smartphone use;*

a *male, 1; female =2*.

**Table 5:** Bootstrapping the conditional direct and indirect effects and 95% confidence interval (CI) for the moderated mediation model.

**Direct path (Academic burnout → PSU)**	**Estimated effect**	**BootLLCI**	**BootULCI**	
%	Moderator: resilience (mean centered)			
16^th^	−13.027	^a^0.262	0.188	0.336
50^th^	−2.027	^a^0.196	0.14	0.252
84^th^	12.973	^a^0.107	0.035	0.179
**Indirect path (Academic burnout → anxiety → PSU)**	**Estimated effect**	**BootLLCI**	**BootULCI**	
%	Moderator: resilience (mean centered)			
16^th^	−13.027	^a^0.04	0.012	0.069
50^th^	−2.027	^a.^0.022	0.002	0.042
84^th^	12.973	^b^–0.008	−0.035	0.016

*N = 748. Bootstrap sample size, 5,000; PSU, Problematic smartphone use; LL, low limit; CI, confidence interval; UL, upper limit;*

a *Empirical 95% confidence interval does not overlap with zero;*

b *Empirical 95% confidence interval overlaps with zero*.

## Discussion

The current study focused on academic burnout, anxiety, resilience, and problematic smartphone use. We found that academic burnout positively predicted problematic smartphone use both directly and indirectly *via* anxiety. More importantly, we identified the role of resilience in moderating the progression of problematic smartphone use.

### The Direct Impact on Problematic Smartphone Use

Supporting Hypothesis 1, we observed that academic burnout positively predicted problematic smartphone use, which concurs with previous findings (28). According to the previous studies (30, 50), academic burnout is linked with the negative emotions of an individual. Consequently, a student with academic burnout would turn to smartphone use for a temporary relief. However, when the tendency becomes out of control, an individual as such would develop problematic smartphone use.

### The Mediation Effect of Anxiety

Supporting Hypothesis 2, our study demonstrated the mediation effect of anxiety between academic burnout and problematic smartphone use, which is in line with the I-PACE model (36, 37). As suggested by previous findings (30, 31), a prolonged state of burnout would lead to a state of anxiety within an individual, and this is also true of the student population. With a higher level of anxiety, a student, if not properly trained to navigate through the challenging situation, would easily depend on the cyber world for support. This scenario increases the chances to develop problematic smartphone use.

### The Moderation Effects of Resilience

Partly supporting Hypothesis 3, the study found that resilience moderated between anxiety and problematic smartphone use and between academic burnout and problematic smartphone use; with a higher level of resilience, the impact of academic burnout on problematic smartphone use and the impact of anxiety on problematic smartphone use both became smaller. Resilience is a positive psychological factor, which enhances the capability of an individual to journey through life adversities and guides an individual back to the normal track (34). Resilience protects individuals against the stressors that may lead to anxiety by shifting their focus to the positive resources, which counteract the influences brought by negative emotions (51). With resilience exerting a positive effect, an individual would actively go with anxiety and be cautious of smartphone use; as a result, the chance to develop problematic smartphone use is reduced. In addition, the enhanced perspectives of the challenging situation and readiness to overcome the difficulties would help a student to stay concentrated on the academic work at hand without being distracted by unnecessary smartphone use. Consequently, with a higher level of resilience, the direct impact of academic burnout on problematic smartphone use would be effectively attenuated.

### Limitations and Implications

The current study had several limitations. First, the current study was based on a cross-sectional study, and the causality of the investigated association could not be built. Second, the imbalanced gender distribution might influence the results.

Despite the limitations, the current study provides some implications. First, we examined the role of anxiety as the mediator between academic burnout and problematic smartphone use. The COVID-19 pandemic has greatly increased the level of psychological distress among college students (2). Besides the direct impact of enhancing the levels of negative emotions, the indirect influence, such as promoting the development of problematic smartphone use, also calls for our attention. With our finding, timely measures should be implemented to take good care of the psychological status of the college students not only for alleviating the direct effect but also for buffering the potential side effects. Second, significantly, our study identified the moderation effects of resilience in alleviating the impact of academic burnout on problematic smartphone use. While pursuing the effective measure to stop the spreading of the virus, it should be clear in all parties involved that the psychological impacts due to the pandemic are also in need of immediate care. Our study has provided the potential solution, and with this, the educational authorities could adjust the syllabus accordingly and emphasize the training of the resilience level of the students, which would help minimize the losses.

## Conclusion

The current study focused on academic burnout, anxiety, resilience, and problematic smartphone use among Chinese undergraduate students during the COVID-19 pandemic. Our study showed that academic burnout predicted problematic smartphone use both directly and indirectly via anxiety. Significantly, we found the moderation effect of resilience in the process. Our findings added evidence to the association between academic burnout and problematic smartphone use and significantly suggested the potential solution to buffer the impacts.

## Data Availability Statement

The datasets generated for this study are available on request to the corresponding author.

## Ethics Statement

The studies involving human participants were reviewed and approved by Liaoning National Normal College. The patients/participants provided their written informed consent to participate in this study.

## Author Contributions

ZH and QC: designed the study and wrote the protocol. ZH, LJ, and JH: conducted literature searches and provided summaries of previous research studies. ZH and RL: conducted the statistical analysis. LJ, JH, and RL: collected the data. ZH: wrote the first draft of the manuscript. All authors contributed to and have approved the final manuscript.

## Funding

This work was supported by the Liaoning Provincial Federation Social Science Circles, China (grant number: 2022lslybkt-051).

## Conflict of Interest

The authors declare that the research was conducted in the absence of any commercial or financial relationships that could be construed as a potential conflict of interest.

## Publisher's Note

All claims expressed in this article are solely those of the authors and do not necessarily represent those of their affiliated organizations, or those of the publisher, the editors and the reviewers. Any product that may be evaluated in this article, or claim that may be made by its manufacturer, is not guaranteed or endorsed by the publisher.
